# A metabolite of leucine (β-hydroxy-β-methylbutyrate) given to sows during pregnancy alters bone development of their newborn offspring by hormonal modulation

**DOI:** 10.1371/journal.pone.0179693

**Published:** 2017-06-15

**Authors:** Tomasz Blicharski, Ewa Tomaszewska, Piotr Dobrowolski, Monika Hułas-Stasiak, Siemowit Muszyński

**Affiliations:** 1 Chair and Department of Rehabilitation and Orthopaedics, Medical University in Lublin, Lublin, Poland; 2 Department of Animal Physiology, Faculty of Veterinary Medicine, University of Life Sciences in Lublin, Lublin, Poland; 3 Department of Comparative Anatomy and Anthropology, Maria Curie-Skłodowska University, Lublin, Poland; 4 Department of Physics, Faculty of Production Engineering, University of Life Sciences in Lublin, Lublin, Poland; INIA, SPAIN

## Abstract

The effects of dietary β-hydroxy-β-methylbutyrate (HMB) supplementation during gestation on bone, growth plate, and articular cartilage in newborns were determined. Thermal analysis of articular cartilage was performed to examine the structural changes in collagen. At day 70 of gestation, a total of 12 sows (Large White Polish breed, at the second parity) were randomly assigned to two groups, with each group receiving either a basal diet or the same diet supplemented with 0.2 g/day HMB until the 90^th^ day. Maternal HMB supplementation enhanced body weight, bone length, and diameter in males. It also improved geometric and mechanical properties contributing to increased bone morphology and endurance. In turn, alteration of the length was only observed in females. The positive effects were mediated by increased serum concentrations of insulin-like growth factor-1 and leptin. HMB-treatment enhanced the concentration of FSH, LH, estradiol, and testosterone. Serum TAP was enhanced by the HMB-treatment by 34% in females and 138% in males. Beneficial effects of the HMB-treatment on trabecular bone and content of proteoglycans in articular cartilage were shown. The HMB-treatment significantly changed the collagen structure in cartilages, especially in the females, which was demonstrated by the PSR analysis. Differences between the HMB-supplemented and the control females in the calorimetric peak temperatures were presumably related to different collagen fibril density in the articular cartilage structure. In summary, maternal HMB supplementation in the mid-gestation period significantly improved general growth and mechanical endurance of long bones by the influence on the somatotropic and pituitary-gonadal axes in the offspring.

## Introduction

Leucine (LEU) is the essential branched-chain amino acid, a potent anti-catabolic compound and regulator of protein metabolism. LEU appears to be unique among branched-chain amino acids with regard to its effectiveness as a nutrient regulator of protein synthesis in skeletal muscle [1]. High LEU doses counteract muscle proteolysis, while low LEU doses enhance muscle protein synthesis [2], which has been proved in numerous *in vivo* and *in vitro* studies [3]. In normal conditions, about 80% of LEU is used for protein synthesis while the rest is converted to alfa-ketoisocaproate, and approximately 5% of leucine is employed in endogenous synthesis of β-hydroxy-β-methylbutyrate (HMB) [2, 4]. As a natural biologically active compound, HMB is also present in many food products such as catfish, alfalfa, asparagus, avocado, or grapefruit [3]. Oral administration of LEU or HMB enhances protein synthesis not only in skeletal muscle but also in other tissues [1, 5]. Moreover, multiple clinical studies show that HMB counteracts protein degradation in many pathological conditions like AIDS trauma bed rest, cancer cachexia, or IUGR [3, 6]. Many *in vitro* studies have been conducted to examine the mechanism of HMB action [3]. Observations in humans, additionally supported by animal studies, explain that some of HMB reparative effects on damaged tissue may be associated with its influence on the growth hormone (GH) and insulin-like growth hormone 1 (IGF-1) axis, which affects myoblast proliferation, differentiation, and survival [2]. Moreover, it has been also demonstrated in animal studies that prenatal administration of HMB has a positive impact on postnatal growth and development [7]. It has been proved that maternal supplementation of HMB affects the physical properties of the enamel surface in spiny mice offspring [8].

There has been increased interest in nutritional prenatal programming in both animal and human research [9–15]. Some changes, e.g. adaptive, functional, structural, and metabolic alterations occurring during prenatal development as an effect of the interaction between genes and different factors, trigger permanent alterations in the homeostasis in developing organism. [12, 13, 15–18]. The structural quality of connective tissue is determined mainly by genes, but it fluctuates with age and health, and comes under the impact of hormonal and nutritional adaptation during the prenatal period in both animals and humans [7, 11–13, 19]. Foetal life is a critical time for the growth and development of systems involved in the pathology of bone metabolism. Various experimental investigations and clinical statistics verify the hypothesis of prenatal programming of many diseases of adults, including osteopenia or osteoporosis [7, 11–13, 19]. Recently, there has been increased interest in HMB in both animal and human research. However, there is an insufficient number of studies concerning beneficial or any effects on both animal and human offspring achieved by addition of HMB to their mothers`nutrition.

The fact that cartilage consists of collagen proteins had encouraged us to perform the thermal analysis to examine the structural changes in collagen following prenatal programming. Numerous articles have illustrated that differential scanning calorimetry (DSC) is a validly efficient method for demonstration of structural changes in different tissue elements in biological systems [20, 21]. Therefore, it should be a suitable method for analysis of thermal consequences of conformational changes in collagen-rich cartilage tissues [22, 23].

The present study was conducted to test the hypothesis that maternal HMB treatment during gestation could positively influence the development of the skeletal system of newborn offspring in a sex-dependent manner. Therefore, the present study involved multiple methods, i.e. mechanical testing, thermal and densitometric analysis, as well as light microscopy in combination with histochemical and immunohistochemical methods to characterize the role of maternal HMB nutrition in foetal bone development. The effects of 20-day long prenatal treatment with HMB on serum insulin-like growth factor-1 (IGF-1), leptin, and the pituitary-gonadal axis in newborn piglets were determined as well. The measurement of total alkaline phosphatase (TAP) activity in blood serum was performed to assess osteoblastic activity. Properties of the skeletal system were investigated in newborn piglets by determination of bone tissue density, geometrical parameters, and mechanical endurance of the femur. Furthermore, the histomorphometry of trabecular bone and articular and growth plate cartilages, the content of thin and thick collagen and proteoglycan, and the expression of osteoprotegerin in the bone were assessed.

## Material and methods

The experiment was approved by The Local Ethics Committee on Animal Experimentation of University of Life Sciences in Lublin, Poland (2014/29).

### Animals, breeding and experimental design

The study was performed on 24 piglets born by 12 sows of Large White Polish breed. Sows were sired by the same boar. Sows were clinically healthy and singly housed in separated cages under standard rearing conditions (controlled temperature, humidity and 12:12-h light-dark cycle) with free access to fresh water and fed twice a day (2.3 kg/day) with balanced standard commercial diet for pregnant and lactating sows commonly used [7]. Primiparous sows were used in the control and experimental groups to avoid the effect of the number of pregnancies on the birth weight in the offspring. The sows in both groups were ca. 10 months old and mated during the third oestrus. Further, during our experiment, were did not observe loss of fat tissue in the pregnant sows in the first trimester of gestation, too slow body weight and fat tissue gains in the pregnant sows, or excess muscle tissue gains in the first trimester of pregnancy, which could have been a cause of IUGR. For this purpose, a two-phase feeding system for pregnant sows was introduced and the composition and amounts of feed were controlled in each phase of gestation.

To investigate the effects of prenatal treatment with a leucine metabolite on cartilage, β-hydroxy-β-methylbutyrate (HMB; Lonza^©^, Basel, Switzerland) was administered to sows in mid-pregnancy. The sows were randomly assigned into two weight and age-matched groups (6 sows in each group). To investigate the detrimental effects of HMB on the articular and growth plate cartilages and bone morphology, the sows were fed with a standard diet (control) and diet supplemented with HMB (0.2 g/kg of body weight/every day) in the morning meals from the 70^th^ to 90^th^ day of gestation. The dose of HMB was determined from a previous study, where we used HMB in a dose of 0.2 g/kg/every day and a significant effect on enamel surface development of the offspring was found at this dose [8]. In the present study, we also decided to perform the HMB treatment during the period from the 70^th^ to 90^th^ day of gestation. Based on our previous studies, it seems that this period covers the most responsive time in prenatal growth in pigs, especially males [24, 25]. The gestation length did not differ between the control and HMB-sows.

All piglets born by physiological partum had no congenital changes. Primiparous sows exhibit 15% lower fertility than multiparous females, thus the average number of piglets born per litter was approx. 10.5±0.5 in the control group and 9.5±1.2 in the HMB group. There were no stillbirths. The placentas did not contain mummified piglets. Females and males used in the study were treated in the same manner. Due to the absence of weight outliers (Grubbs statistics), 6 individuals of each sex were drawn from each group using a random number generator. Piglets born by sows fed with the control diet belonged to the control group of males (n = 6) and females (n = 6), and piglets born by HMB-treated sows belonged to the HMB group of males (n = 6) and females (n = 6). At birth, unsuckled piglets were weighed and euthanized by intravenous injections of lethal doses of pentobarbitalum natrium (Morbital, Biowet, Puławy, Poland). Basal vital organs such as liver, heart, lungs, spleen, stomach, kidneys, and brain were isolated and weighted. Since the experiment was conducted on newborns, there was no need for translocation of the piglets between mothers or elimination of boars (to equalise the number of piglets in the litters or obtain the best females as a maternal material).

### Geometric parameters

After removal of soft tissues, left femora were used for bone length and weight measurement. Each bone was stored at -25°C for further analysis after wrapping in gauze soaked in isotonic saline. The cross-section area (A), mean relative wall thickness, and cortical index (CI; defined as the ratio of the thickness of cortical part to the thickness of the midshaft measured in the middle part of the bone) were calculated on the basis of horizontal and vertical diameter measurements of the midshaft cross-section [26–28].

### Mechanical properties

Each femur was thawed for 3hours at room temperature and the mechanical properties were determined using the three-point bending test. The test was performed on a universal testing machine (Zwick Z010, Zwick GmbH & Company KG, Ulm, Germany), equipped with TestXpert II 3.1 software (Zwick GmbH & Company KG, Ulm, Germany). The relationship between force exerted perpendicularly to the longitudinal axis of the bone and the resulting displacement was registered. The supports were placed at 40% of the total bone length. The measuring head loaded bone samples with a constant speed of 10 mm/min until fracture as it was described previously [29]. The ultimate endurance was determined as the force causing bone fracture and the maximum elastic strength as maximal force under elastic (reversible) deformation of bone [27, 30]. The registered strength data were also normalized to bone geometry and expressed as a ratio of recorded force and the mid-diaphysis cross section area. Moreover, on the basis of measured geometric and mechanical traits the material properties of the mid-diaphyseal fragment of the bone were calculated with method described previously [30]. These traits describe the specific mechanical properties of midshaft cortical tissue and are independent of the bone size and the conditions under which the strength tests were conducted. Bending moment can be described as a yield load adjusted to the bone length and it indicates bone elastic load capability [30]. The elastic stress reflects the elastic strength of midshaft cortical bone, the ultimate stress is equal to the maximum stress a bone can withstand in bending before fracture [30, 31].

### Histomorphometry

Only the joints without visible lesions and other changes were opened. Soft tissues were removed and full-thickness cartilage was excised. 20-mm thick cylindrical samples (including cartilage and bone) were obtained from the middle of the lateral condyle (containing epiphysis and metaphysis). Sagittal sections of the samples were cut perpendicularly to the articular surface [32]. The bone samples were subjected to common histological and microscopic procedures [32–34]. 4-μm thick sections were cut. Goldner’s trichrome staining was performed to evaluate basal morphology of the growth plate and articular cartilages [34]. Safranine O (SO) was used to stain proteoglycans in articular cartilage [34, 35]. Microscopic images were collected in brightfield using a confocal microscope (Axiovert 200M, Carl Zeiss, Jena, Germany) equipped with a camera (AxioCam HRc, Carl Zeiss, Jena, Germany) and a halogen lamp.

Picrosirus red staining (PSR) was employed to assess the morphology of articular cartilage and to evaluate the distribution of thick and thin collagen fibres in articular cartilage [24, 34, 36, 37]. Sections were analysed using a microscope (Olympus BX63; Olympus, Tokyo, Japan) equipped with filters providing circularly polarized illumination (the filters were aligned so that the background in the field of view was as dark as possible, i.e. the filters were “crossed”). Images were collected with a CDD camera (UC50 Olympus, Tokyo, Japan).

The analysis of the received images was performed using graphical software Olympus cellSens Version 1.5 (Olympus, Tokyo, Japan). The thickness of main zones of the growth plate was measured at four sites and an average was calculated. In the reserve zone (I), cells exist singly or in pairs and are separated by an abundant extracellular matrix. In the proliferative zone (II), flattened chondrocytes are arranged in longitudinal columns and they enlarge and divide. In the hypertrophic zone (III), cells abruptly increase the size and the columnar arrangement is irregular. The zone of ossification (IV) is the region of cartilage transition to bone. Degeneration and death of chondrocytes occur there [32, 33]. The thickness of the main zones of the articular cartilage, i.e. superficial surface (horizontal I; small chondrocytes are flattened parallel to the surface), transitional (II; large and round chondrocytes occur singly or in isogenous groups), radial (III; columns of spherical chondrocytes) was measured [26, 38].

The bone (BV) and tissue volumes (TV) were measured in the images of the tissue sections using the pixel count, and the relative bone volume (BV/TV%) was calculated. The following parameters for trabecular bone (epiphysis and metaphysis) were examined: trabecular thickness (Tb.Th), trabecular separation (Tb.Sp; defined as the distance between the edges of adjacent trabeculae, measured directly), and trabecular number (Tb.N) [26, 38, 39].

### Bone and cartilage tissue density

Bone metabolism was assessed by determining bone mineral density (BMD) and bone mineral content (BMC) for the whole bone using the dual-energy X-ray absorptiometry (DEXA) method on a Discovery W Hologic X-ray densitometer (Bedford, MA, USA) and APEX 3.0.1 software with a Small Animals Studies option for investigation of bones from various types of animals. Densitometer self-calibration using the Hologic Automatic Internal Reference System was performed before measurements. An optional Scout scan was available to assist the operator in defining the scan region. The analysis was performed on the scan data using an operator–defined region of interest and numeric results were calculated and displayed. The operator had the capability of adjusting the start and the end points of the Scout and Measurement scan. The measurements of BMC and BMD were performed using the following parameters: additional calibration with Hologic rat step phantom P/N 010–0758 Rev.004 and the resolution 0.5 line pair/mm (1.0x1.0 mm). The region of interest after the scout scan was defined manually.

The measurement of bone tissue density (BTD) of distal articular cartilage was performed with a helium gas pycnometer (AccuPyc 1330; Micromeritics, Gosford, USA) equipped with a 10 cm^3^ metal measuring cylinder as it was described [40].

### Immunohistochemistry

Osteoprotegrin (OPG), an osteoclastogenesis inhibitory factor, was determined in trabecular bone. Immunohistochemical staining of decalcified serial sections was performed according to a protocol described previously [26, 41].

Briefly, after deparaffinization and rehydration with distilled water, antigen retrieval was achieved by 10-min enzymatic retrieval with proteinase K (Sigma, Poland) in 37°C. Endogenous peroxidase activity was blocked subsequently with a 3% solution of hydrogen peroxide in methanol (1:1) for 30 min. After blocking in normal serum, sections were incubated with the first antibody over night at 4°C. Rabbit polyclonal to osteoprotegerin (OPG) antibodies (Abcam, Cambridge, UK, dilution 1:100) were used as first antibodies. The sections were then incubated (30 min) with the second antibody: biotinylated anti-rabbit immunoglobulin (Abcam, Cambridge, UK, dilution 1:200). Negative control sections for each antibody were obtained by identical immunohistochemical staining excluding the primary antibody. Then the sections were developed in DAB (DacoCytomation); 30,30-diaminobenzidine tetrahydrochloride was used as a chromogene for 15 min at room temperature. Counterstaining was performed with hematoxylin [26, 41].

Microscopic observations and images of immunohistochemistry reactions were further analysed. The expression of OPG was described in trabecular osteocytes and growth plate as positive (brown nuclei) or negative (blue nuclei) reaction [26, 41].

### Measurement of cartilage and bone turnover markers

Each newborn piglets was unsuckled. The animal blood was collected using standard venepuncture; next, after clotting at room temperature, it was centrifuged and frozen at -80°C for further analysis. All the samples were determined in duplicate.

Changes in the somatotropin axis were assessed by determination of insulin-like growth factor-1, which stimulates proliferation and differentiation of osteoblasts, enhances synthesis of collagen type I, and increases the activity of BAP and osteocalcin production. The concentration of insulin-like growth factor-1 (IGF-1) using Porcine IGF-1 Enzyme-Linked Immunosorbent Assay Kit (Uscn Life Science Inc.Wuhan, P. R. China). The threshold detection value was 34 pg/mL.

As an important growth factor in intrauterine and neonatal development leptin concentration was determined with the use of Porcine Leptin Enzyme-Linked Immunosorbent Assay Kit (Uscn Life Science Inc.Wuhan, P. R. China). The minimum detection was 12.7pg/mL.

The concentration of porcine luteotropic hormone was determined by the use of LH ELISA Kit (Shanghai Sunred Biological Technology Co. Baoshan District, Shanghai). The minimum detection was 0.038 ng/mL. The concentration of porcine follicle-stimulating hormone was determined by the use of FSH ELISA Kit Shanghai Sunred Biological Technology Co. Baoshan District, Shanghai). The minimum detection was 0.745 IU/L. Estradiol concentration was determined used an Porcine 17β-estradiol ELISA Kit (Southern California, San Diego, USA). The minimum detection was 0.06 ng/mL. Testosterone concentration was determined used an Porcine Free testosterone ELISA Kit (Southern California, San Diego, USA). The minimum detection was 0.05 ng/mL.

Serum hormonal concentrations were assessed by means of a Benchmark Plus microplate spectrophotometer (Bio-Rad Laboratories, Inc., Hercules, CA, USA). Serum concentration of selected hormones was determined using commercially available kits.

### Differential scanning calorimetry (DSC) analysis

After the dissection, cartilage samples obtained from femoral condyle were washed three times in distilled water in order to eliminate tissue remnants. All the samples were put into 0.9% sodium chloride solution and stored separately at 7°C, no longer than 24 h, before they were subjected to DSC measurements. Prior the measurements, cartilage were removed from PhS and gently patted dry superficially on filter paper. Specimens were trimmed with a razor blade to fill the DSC pan and ensure contact with the pan for optimal heat flux. Typical cartilage wet weights were between 4 and 7 mg. DSC was performed using a differential scanning calorimeter (Model DSC-1, Mettler-Toledo GmbH, Switzerland). Temperature and heat flow scales were calibrated with indium standards. Specimens were hermetically sealed in 40 μl aluminum pans to prevent any loss of moisture during measurements and run against an empty pan for reference. Heating was carried out at 10°C/min from 20°C to 85°C under the nitrogen flow (50 ml/min). For each endothermal process obtained on the thermogram, the initial (onset) temperature (T_on_), peak temperature at maximum heat absorption (T_max_) corresponding to denaturation temperature for each sample were determined using a software integrated with the calorimeter (STAR^e^ Evaluation software ver. 15, Mettler-Toledo GmbH, Switzerland). Following calorimetry, the pans were punctured and the samples dried for 24 h in an oven at 105°C for determination of the dry mass. Water content was in the range from 83 to 86% and the differences between the samples were not statistically significant.

### Blood serum biochemical analyses

The blood serum immediately separated by centrifugation was stored at -25°C for further analysis. The serum total alkaline phosphatase (TAP) activity, phosphorus, ionized calcium, and total calcium were assessed photocolorimetrically (Metrolab 2300 GL, Metrolab SA, Buenos Aires, Argentina) and with ready-made sets (BioMaxima, Lublin, Poland).

### Statistical analysis

The statistical analysis of results was performed with the software package Statistica 12.0 (Statsoft Inc., Tulsa, OK, USA) following a general linear model. The statistical model included the variables supplementation and offspring’s sex as well as interactions between supplementation and sex. Means values were compared by Tukey’s multiple comparison test, the probability level of p<0.05 was considered as statistically significant.

## Results

### Body and organ weight

The maternal HMB treatment enhanced body weight (Table 1), but this effect was evident only in the male newborn offspring (an increase of 81% compared to the male control newborns). Moreover, HMB given to sows during pregnancy increased the weight of the liver, lung, and stomach. The maternal HMB treatment influenced also the weight the brain, but it was statistically significant only in male newborns, compared to female newborns in general. Further, the HMB supplementation did not influence the weight of the spleen (Table 1).

**Table 1:** The effect of maternal treatment with HMB on body, bone and organs weight, the length of the femur and mass/length ratio in newborn piglets.

Item	Female		Male		SEM	Influence of		
	control	HMB	control	HMB		treatment	sex	Interaction of treatment*sex
**Body weight, g**	884^a^	1267^ab^	922^a^	1675^b^	72	<0.001	0.036	0.070
**Liver, g**	17.94^a^	42.92^b^	18.97^a^	52.02^b^	4.00 0.66	<0.001	0.253	0.247
**Heart, g**	6.44^a^	9.92^ab^	6.32^a^	11.62^b^	0.66	0.001	0.211	0.179
**Lung, g**	11.02^a^	19.74^b^	10.14^a^	25.89^b^	1.62	<0.001	0.089	0.033
**Spleen, g**	0.75	1.22	0.75	1.33	0.10	0.011	0.597	0.584
**Stomach, g**	5.53^a^	8.31^bc^	5.56^ab^	10.11^c^	0,53	<0.001	0.124	0.134
**Kidney, g**	7.70^ab^	10.51^b^	5.97^a^	11.63^b^	0.63	<0.001	0.722	0.115
**Brain, g**	33.46^a^	33.88^a^	33.63^ab^	36.85^b^	0.43	0.021	0.044	0.071
**Bone weight, g**	4.61^a^	7.04^ab^	4.93^a^	7.75^b^	0.34	<0.001	0.069	0.452
**Bone length, mm**	44.99^a^	52.73^b^	42.91^a^	53.91^b^	1.05	<0.001	0.303	0.901
**Relative bone weight, %**	0.47^a^	0.58^a^	0.54^a^	0.47^a^	0.03	0.662	0.943	0.379
**Mass/length bone ratio**	0.098^a^	0.122^ab^	0.115^a^	0.146^b^	0.005	0.001	0.031	0.381

^a, b, c^—mean values in rows with different letters differ significantly at P<0.05; SEM—standard error of the means.

### Biochemical analysis

The concentration of phosphorus, ionized calcium, and total calcium in blood serum decreased after the maternal HMB treatment irrespective of the sex of the piglets. Serum TAP increased only in male newborns as a result of the HMB supplementation in their mothers. The concentrations of leptin and IGF-1 increased after the maternal HMB treatment irrespective of the sex of the piglets. The increase in LH was observed only in males, but FSH, estradiol, and testosterone were enhanced in both sexes as a result of HMB given to the sows during pregnancy (Table 2).

**Table 2:** The effect of maternal treatment with HMB on biochemical and hormonal analysis in blood serum in newborn piglets.

Item	Female		Male		SEM	Influence of		
	control	HMB	control	HMB		treatment	sex	Interaction of treatment*sex
**P, mmol/L**	2.38^a^	1.43^b^	2.34^a^	1.62^b^	0.10	<0.001	0.522	0.152
**Ca**^**+2**^**, mmol/L**	1.35^b^	1.24^a^	1.34^ab^	1.11^c^	0.03	<0.001	0.023	0.063
**Total Ca, mmol/L**	2.64^c^	2.41^ab^	2.62^bc^	2.19^a^	0.05	<0.001	0.046	0.101
**TAP, U/L**	2276^ab^	3054^b^	1250^a^	2977^b^	200	<0.001	0.058	0.203
**Leptin, ng/mL**	29.72^a^	39.47^b^	29.84^a^	41.77^b^	0.95	<0.001	0.804	0.171
**IGF I, ng/mL**	22.82^a^	60.70^b^	13.60^a^	53.92^b^	2.98	<0.001	0.029	0.683
**LH, ng/mL**	1.05^a^	1.35^a^	1.05^a^	3.13^b^	0.14	<0.001	0.002	0.002
**FSH, ng/mL**	23.00^a^	52.31^b^	25.96^a^	75.53^c^	4.53	<0.001	0.010	0.039
**Estradiol, ng/mL**	0.881^a^	1.961^b^	0.550^a^	2.255^b^	0.132	<0.001	0.924	0.102
**Testosterone, ng/mL**	0.092^a^	0.123^b^	0.120^ab^	0.170^c^	0.006	<0.001	<0.001	0.317

^a, b, c^—mean values in rows with different letters differ significantly at P<0.05; SEM—standard error of the means.

### General bone properties

**Fig 1:**
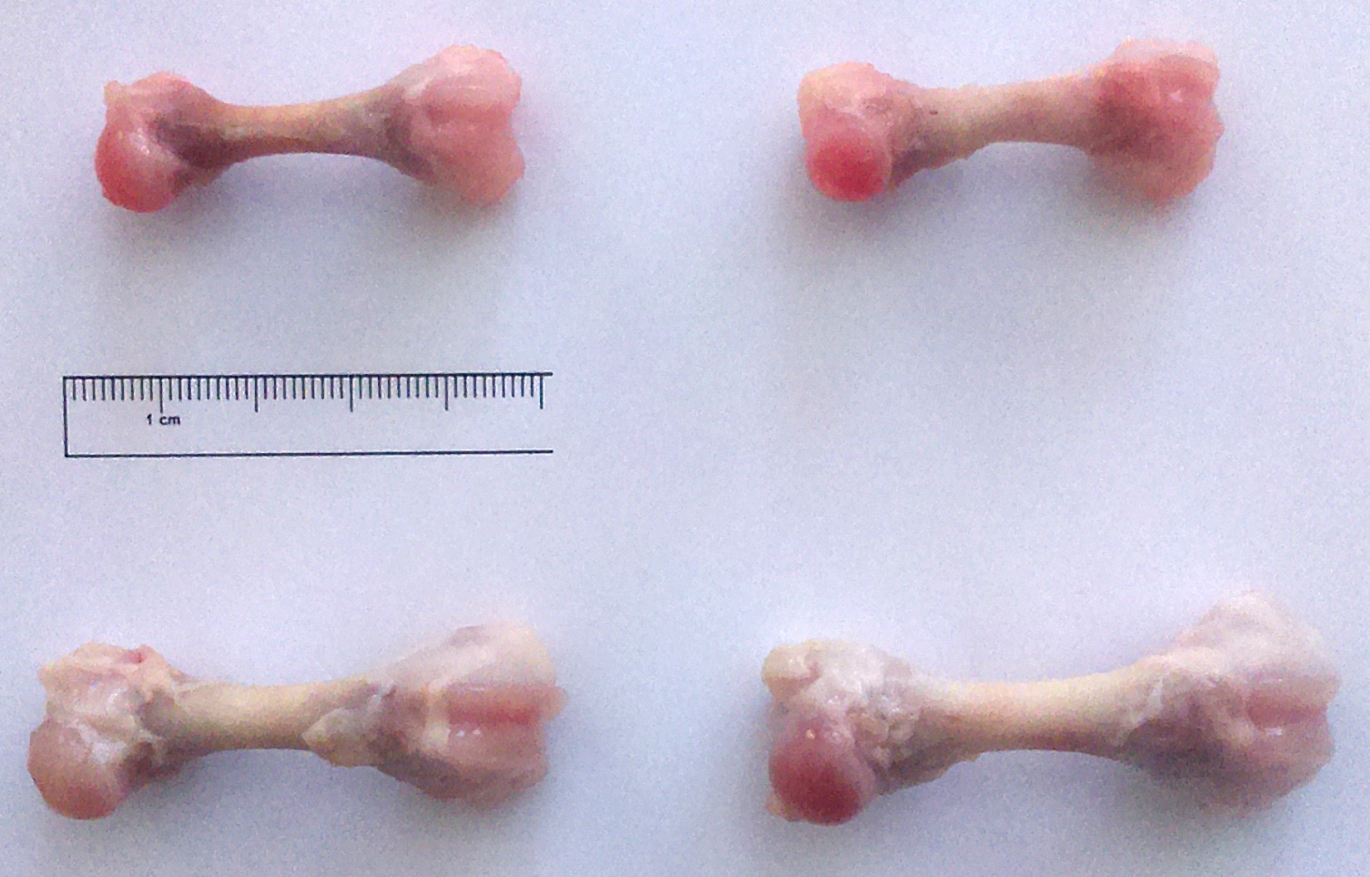
Effects of HMB treatment during prenatal time on the femur morphology in newborn piglets. Upper left corner—femur of the control female newborns; bottom left corner—femur of the HMB-treated female newborns; upper right corner—femur of the control male newborns; bottom right corner—femur of the HMB-treated male newborns.

The maternal HMB supplementation increased bone weight (Table 1); however, this effect was statistically significant only in the male newborn offspring (an increase of 57%). This kind of supplementation also influenced the length of the femur in both sexes, and a longer femur was noted in the HMB groups compared to the control offspring (an increase of 17% and 25% for the males and females, respectively). It also resulted in an increase in the bone to mass ratio of the male offspring (Table 1). However, when the bone mass was compared to newborn piglet’s body weight (bone relative mass), the supplementation turned out not to have influenced bone contribution in body weight neither in the female nor male offspring (Table 1; Fig 1).

### Bone densitometry

Bone mineral content increased after the maternal HMB treatment irrespective of the sex of piglets, while bone mineral density was significantly higher only in male newborns from the HMB group, compared to the female control group (Table 3).

**Table 3:** The effect of maternal treatment with HMB on densitometry, geometric and mechanical properties of the femur in newborn piglets.

Item	Female		Male		SEM	Influence of		
	control	HMB	control	HMB		treatment	sex	Interaction of treatment*sex
*Bone geometric properties*								
**Horizontal internal diameter h, mm (mm)**	2.69	2.36	2.60	2.09	0.09	0.080	0.671	0.559
**Horizontal external diameter H, mm**	5.21^a^	5.46^a^	4.93^b^	5.68^a^	0.09	0.011	0.521	0.361
**Vertical internal diameter b, mm**	2.80	2.49	3.17	2.62	0.11	0.183	0.191	0.455
**Vertical external diameter B, mm**	5.58	5.65	5.13	5.82	0.11	0.094	0.958	0.387
**Cross section area A, mm**^**2**^	17.06^a^	19.50^ab^	13.55^a^	21.66^b^	0.80	0.001	0.889	0.106
**Mean relative wall thickness**	1.02^ab^	1.37^ab^	0.76^a^	1.53^b^	0.08	0.005	0.629	0.202
**Cortical index CI, %**	49.53^ab^	56.49^a^	42.60^b^	58.95^a^	1.57	0.001	0.382	0.113
**Midshaft volume, mm**^**3**^	3.04^ab^	4.11^bc^	2.35^a^	4.72^c^	0.23	<0.001	0.420	0.125
**Moment of inertia Ix, mm**^**4**^	44.30	46.85	30.37	53.70	3.15	0.042	0.923	0.237
**Index of gyration Rg, mm**	1.56	1.54	1.46	1.57	0.03	0.413	0.926	0.620
*Bone mechanical properties*								
**Ultimate strength, N**	168^ab^	214^ab^	141^a^	240^b^	11	0.002	0.752	0.304
**Max. elastic strength, N**	147^a^	151^ab^	107^a^	190^b^	7	0.002	0.576	0.008
**Elastic stress, MPa**	44.48	49.22	40.29	56.65	1.76	0.016	0.531	0.126
**Ultimate stress, MPa**	50.31	69.03	52.99	70.87	2.62	0.006	0.624	0.931
**Bending moment, N·m**	0.649^ab^	0.790^b^	0.463^a^	1.036^c^	0.048	<0.001	0.596	<0.001
*Bone densitometry*								
**Bone mineral content BMC, g**	0.430^a^	0.787^b^	0.442^a^	0.873^b^	0.070	0.005	0.683	0.754
**Bone mineral density BMD, g/cm**^**2**^	0.169^a^	0.206^ab^	0.171^ab^	0.235^b^	0.009	0.002	0.278	0.341

^a, b, c^—mean values in rows with different letters differ significantly at P<0.05; SEM—standard error of the means.

### Bone geometrical properties

The maternal HMB supplementation did not influence the values of the internal (h) as well as the vertical external (B) and internal (b) diameters of the mid-diaphyseal cross-section of the femur in both sexes (Table 3). However, an increase in the horizontal external (H) diameter was observed in the male newborns compared to the control male group (an increase of 15%). As a result, although the supplementation generally influenced bone geometry, a statistically significant increase in several calculated indices characterizing the midshaft wall thickness (cross-sectional area, MRWT) and traits indicating bone filling with cortical tissue (cortical index, midshaft volume) was observed only in the male HMB group, compared to the corresponding control (Table 3). However, due to the large standard deviations, the overall values of the cross-sectional moment of inertia (Ix) were not statistically different between all experimental groups, even if there was a significant influence of the treatment (p<0.043). Similarly, the radius of gyration Rg did not differ between the groups (Table 3).

### Bone mechanical properties

The maternal HMB supplementation resulted in a significant increase in maximum elastic strength in the male offspring (an increase of 77%; Table 3). Similarly, the bending moment was significantly increased in the male HMB group (an increase of 123%). The value of ultimate strength was also influenced by the maternal HMB treatment in the male group (increase of 79%). In the female group, there was only a tendency towards an increasing value of the ultimate endurance in the HMB group, but the analysis showed that this increase was not statistically significant (Table 3). Similarly, although the influence of the supplementation was also significant in two material properties of the femur, namely elastic stress and ultimate stress (p = 0.016 and p = 0.006, respectively), the mean values of this traits did not differ significantly between the groups. These results indicated that, although the male HMB group was characterized by greater mechanical endurance than the control group, stresses that occurred in the bones during deformation were at the same level in all experimental groups (Table 3).

#### Bone histomorphometry

The histomorphometric analysis revealed significantly lower values of the relative bone volume in femoral epiphysis in the male newborns, even after the HMB-treatment, compared to females in general. However, an increase in the relative bone volume and trabecular thickness was observed after the HMB-treatment irrespective of the sex (Table 4). On the other hand, the trabecular space decreased as a result the maternal HMB-treatment. The number of trabeculae in the epiphysis increased in the females from the HMB group, compared to the other groups (Table 4). Moreover, the maternal supplementation with HMB increased the bone volume as well as trabecular thickness and number, and decreased trabecular space in the metaphysis only in female newborns (Table 4).

**Table 4:** The effect of maternal treatment with HMB on trabecular bone morphology of the femur obtained from newborn piglets.

Item	Female		Male		SEM	Influence of		
	Control	HMB	Control	HMB		treatment	sex	Interaction of treatment*sex
*Femur epiphysis*								
**BV/T, %**	22.2^b^	39.6^d^	16.2^a^	32.0^c^	1.5	<0.001	<0.001	0.379
**TbTh mean, μm**	51.3^b^	69.7^c^	41.1^a^	69.4^c^	2.1	<0.001	0.001	0.002
**TbTs mean, μm**	173.2^b^	110.0^a^	249.0^c^	150.3^ab^	10.1	<0.001	<0.001	0.165
**Tb.N/mm of bone**	43.1^a^	56.9^b^	39.5^a^	46.7^a^	1.4	<0.001	0.001	0.104
*Femur metaphysis*								
**BV/TV, %**	21.2^a^	38.7^b^	23.3^a^	25.7^a^	1.3	<0.001	0.002	<0.001
**TbTh mean, μm**	57.1^a^	85.6^b^	51.9^a^	59.9^a^	3.2	<0.001	0.003	0.047
**TbTs mean, μm**	212.1^c^	154.0^a^	194.2^bc^	165.7^ab^	6.1	<0.001	0.759	0.146
**Tb.N/mm of bone**	37.5^a^	46.7^b^	46.1^ab^	43.9^ab^	1.3	0.156	0.234	0.021

^a, b, c, d^—mean values in rows with different letters differ significantly at P<0.05; SEM—standard error of the means.

Although the BV/TV and trabecular thickness in femoral epiphysis were in general lower in male piglets compared to females, an increase was observed in both sexes (Table 4). In turn, the trabecular separation was greater in male piglets compared to females, and the increase was observed in both sexes. However, the maternal HMB treatment resulted in an increase in the number of trabeculae in epiphysis only in the female piglets (Table 4). The supplementation with HMB in pregnant sows also resulted in enhanced BV/TV, number of trabeculae, and thickness in metaphysis only in the female newborn offspring, in which a decrease in trabecular separation was additionally observed (Table 4).

### Morphology of articular and growth plate cartilages

The articular cartilage in the control female piglets was thinner compared to the control male newborns, but an increase in its total thickness was noted after the maternal treatment (Table 5). Further, the thickness of zone I was lower in the control male piglets compared to the control females, but HMB given during the prenatal time enhanced its thickness in both sexes (Table 5). In general, zone II was thinner in the female piglets, compared to the males, but its thickness increased in both sexes after the HMB treatment. However, different results were noted with regard to zone III, which was thinner in the control males compared to the control females. After the maternal HMB supplementation, elongation was noted in the female offspring, and shortening was evident in the male offspring (Table 5).

**Table 5:** The effect of maternal treatment with HMB on femoral cartilage morphology and densitometry in newborn piglets.

Item	Female		Male		SEM	Influence of		
	control	HMB	control	HMB		treatment	sex	Interaction of treatment*sex
*Femoral articular cartilage*								
**Total, μm**	1383^a^	1898^b^	1921^b^	1904^b^	26	<0.001	<0.001	<0.001
**Zone I, μm**	41^b^	50^c^	18^a^	44^bc^	1	<0.001	<0.001	<0.001
**Zone II, μm**	286^b^	244^a^	585^d^	424^c^	13	<0.001	<0.001	<0.001
**Zone III, μm**	699^a^	1397^d^	1130^c^	891^b^	26	<0.001	0.040	<0.001
*Cartilage densitometry*								
**BTD distal, g/cm**^**3**^	2.52^ab^	2.32^ab^	2.65^a^	2.23^b^	0.05	0.003	0.852	0.226
*Growth plate cartilage*								
**Total, μm**	1148^a^	1746^d^	1273^b^	1600^c^	26	<0.001	0.884	<0.001
**Zone I, μm**	384	872^d^	464^b^	602^c^	18	<0.001	<0.001	<0.001
**Zone II, μm**	163^a^	489^d^	252^b^	409^c^	13	<0.001	0.366	<0.001
**Zone III, μm**	179^a^	191^a^	187^a^	272^b^	5	<0.001	<0.001	<0.001
**Zone IV, μm**	182^a^	457^b^	171^a^	471^b^	15	<0.001	0.910	0.394

^a, b, c, d^—mean values in rows with different letters differ significantly at P<0.05; SEM—standard error of the means.

The total thickness of growth plate, zone I, and zone II was greater in the control males than in the control female piglets, and an increase in its thickness was observed in both sexes after the HMB treatment (Table 5). The effect of the HMB supplementation during pregnancy in sows on zone III was dependent on the sex, and an increase was noted only in the male offspring. Moreover, the maternal HMB treatment resulted in elongation of zone IV irrespective of the sex (Table 5).

### Osteoprotegerin expression in trabeculae and growth plate cartilage

**Fig 2:**
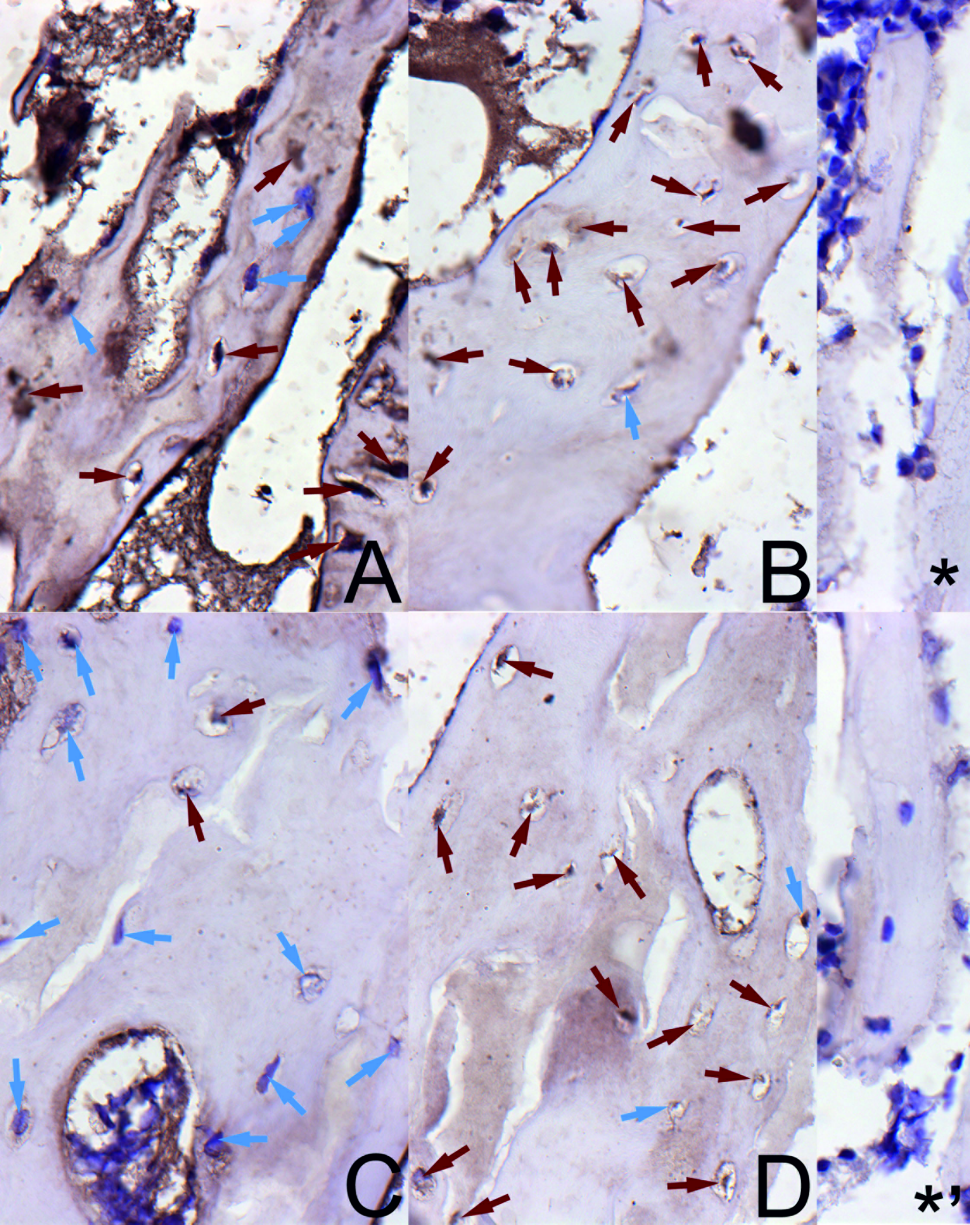
The immunohistochemical analysis of the expression of osteoprotegerin (OPG) from the femoral trabeculae. Representative pictures of the immunohistochemical analysis carried out on formaldehyde-fixed sections from the femoral trabeculae of newborn piglets from the control male group (A), the HMB-treated male group (B), the control female group (C), and the HMB-treated female group (D). * antibody control in males; *^,^ antibody control in females. The nucleus in the chondrocyte with the negative OCN signal is stained blue (blue arrows). The positive OCN signal is indicated in cells by brown arrows. Magnification x200.

The staining for OPG (Fig 2) exhibited low intensity in trabecular osteocytes from the control group in a very small number of osteocytes in both sexes. In many osteocytes in bone trabeculae, violet nuclei without positive reaction for OPG were observed. In turn, the staining for OPG was highly intense or moderate in the trabecular osteocytes from the HMB group irrespective of the sex (Fig 2).

**Fig 3:**
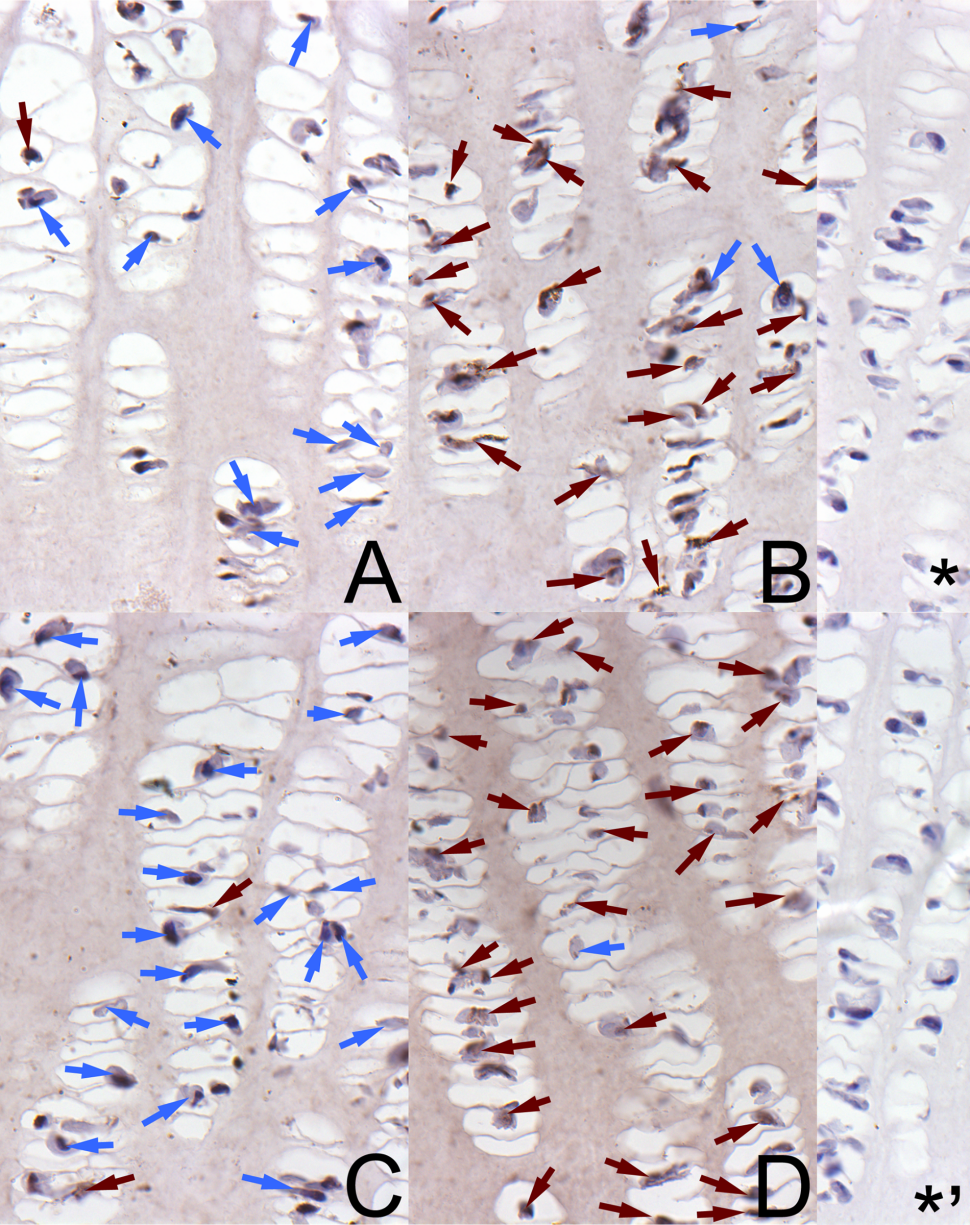
The immunohistochemical analysis of the expression of osteoprotegerin from the proliferative zone of growth plate. Representative pictures of the immunohistochemical analysis carried out on formaldehyde-fixed sections from the femoral growth plate of newborn piglets from the proliferative zone in the control male group (A), the HMB-treated male group (B), the control female group (C), and the HMB-treated female group (D). * antibody control in males; *^,^ antibody control in females. The nucleus in the chondrocyte with the negative OCN signal is stained blue and indicated by blue arrows. The positive OCN signal is indicated in cells by brown arrows. Magnification x200.

**Fig 4:**
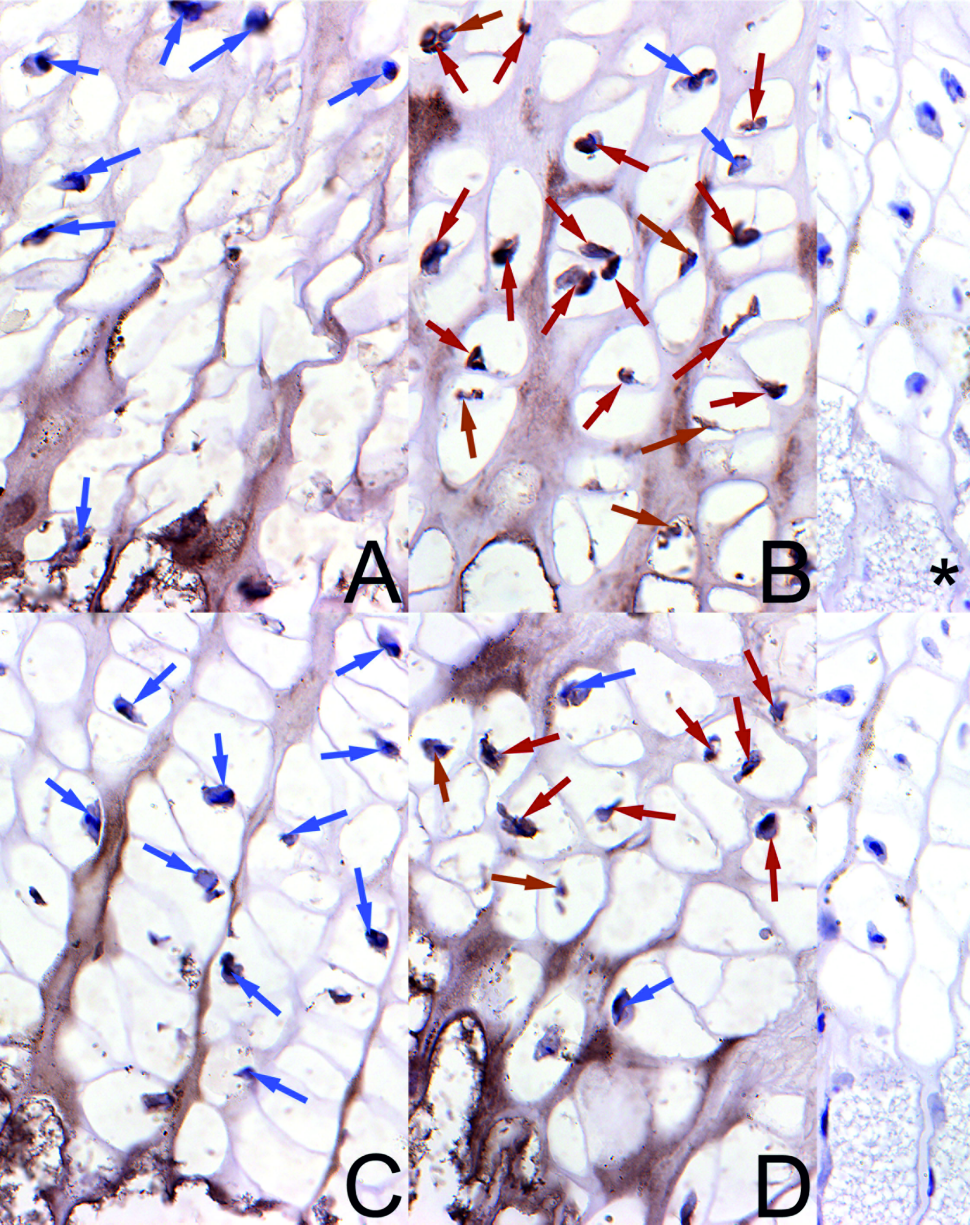
The immunohistochemical analysis of the expression of osteoprotegerin from the hypertrophic zone of growth plate. Representative pictures of the immunohistochemical analysis carried out on formaldehyde-fixed sections from the femoral growth plate of newborn piglets from the hypertrophic zone in the control male group (A), the HMB-treated male group (B), the control female group (C), and the HMB-treated female group (D). * antibody control in males; *^,^ antibody control in females.The nucleus in the chondrocyte with the negative OCN signal is stained blue and indicated by blue arrows. The positive OCN signal is indicated in cells by brown arrows. Magnification x200.

A well-marked brown cytoplasmic signal for OPG in the growth plate was found in the control newborns of both sexes, mainly in chondrocytes from the proliferative zone (Fig 3) and in a very small number of cells from the hypertrophic zone (Fig 4). The reserve and ossification zones were free from the reaction in the control animals. In turn, the maternal HMB treatment resulted in intensive staining of many chondrocytes for OPG in the proliferative zone (Fig 3), and a well-marked cytoplasmic signal was observed in a greater part of the chondrocytes in the hypertrophic zone of the growth plate (Fig 4). The reserve and ossification zones also were free from the reaction.

### Distribution of thick and thin collagen fibres and proteoglycans in articular cartilage

**Fig 5:**
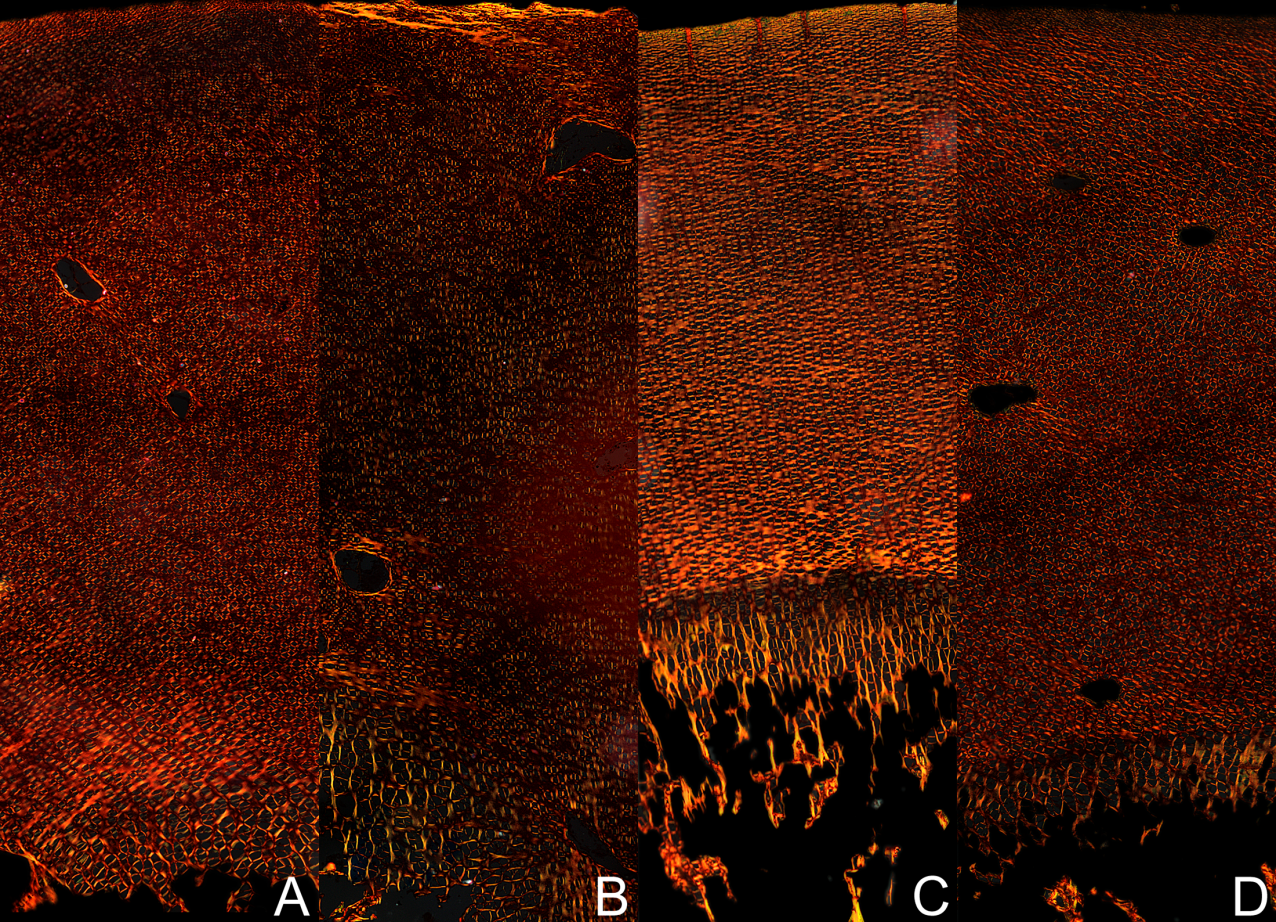
Representative images of femoral articular cartilage stained with PSR carried out on formaldehyde-fixed sections. The large collagen fibres are orange or red and the thick ones including reticular fibres are green. Vertical section of total femoral articular cartilage from the control male group (A), the HMB-treated male group (B), the control female group (C), and the HMB-treated female group (D). Magnification x200.

**Fig 6:**
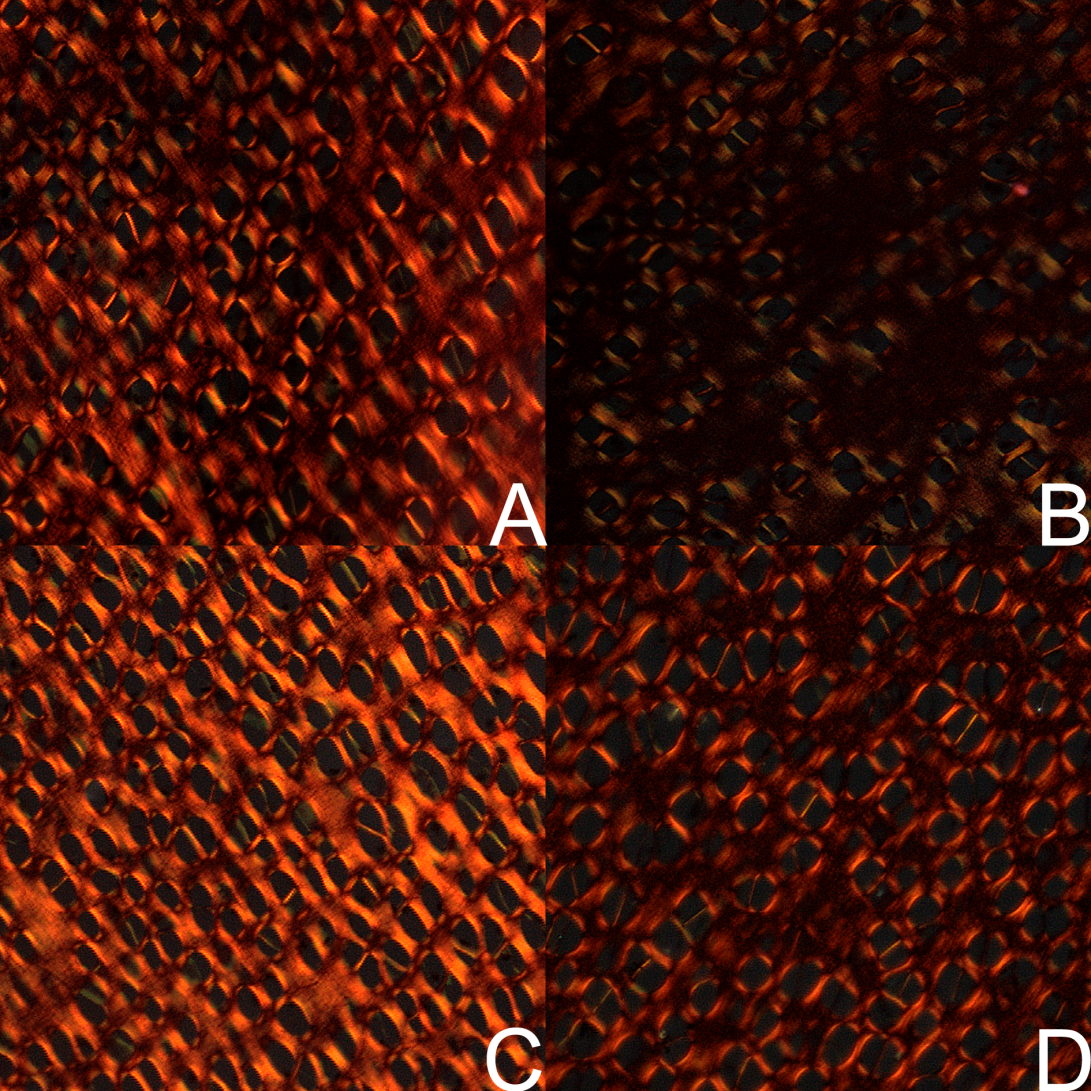
Representative images of PSR staining of transitional zone from femoral articular cartilage. The large collagen fibres are orange or red and the thick ones including reticular fibres are green. Vertical section of the transitional zone of femoral articular cartilage from the control male group (A), the HMB-treated male group (B), the control female group (C), and the HMB-treated female group (D). Magnification x200.

The structural information obtained from the analysis of fibrous components in the PSR-stained section revealed a difference between large (red-orange) and thin collagen fibres including reticular fibres (green). The supplementation of HMB to pregnant sows enhanced thin (green) and decreased thick (red) fibres in articular cartilage resulting in greener transitional and radial fibres around chondrocytes in both sexes, which were more intensive in male piglets (Fig 5). Moreover, fine collagen fibres (green) were distinctly discernible in the layer located near calcified cartilage at the cartilage-bone interface in the HMB-treated male piglets. An opposite proportion was observed in the control newborn pigs, where primarily dark and bright red thick fibres were present in the transitional and radial zones. The HMB-treatment resulted also in a lower amount of loosely packed thin (green) fibres in the total articular cartilage in the female piglets (Fig 6). Moreover, the control cartilage from both sexes of animals and the HMB-treated males contained compacted large (red-orange) fibres in the total articular cartilage (Fig 6).

**Fig 7:**
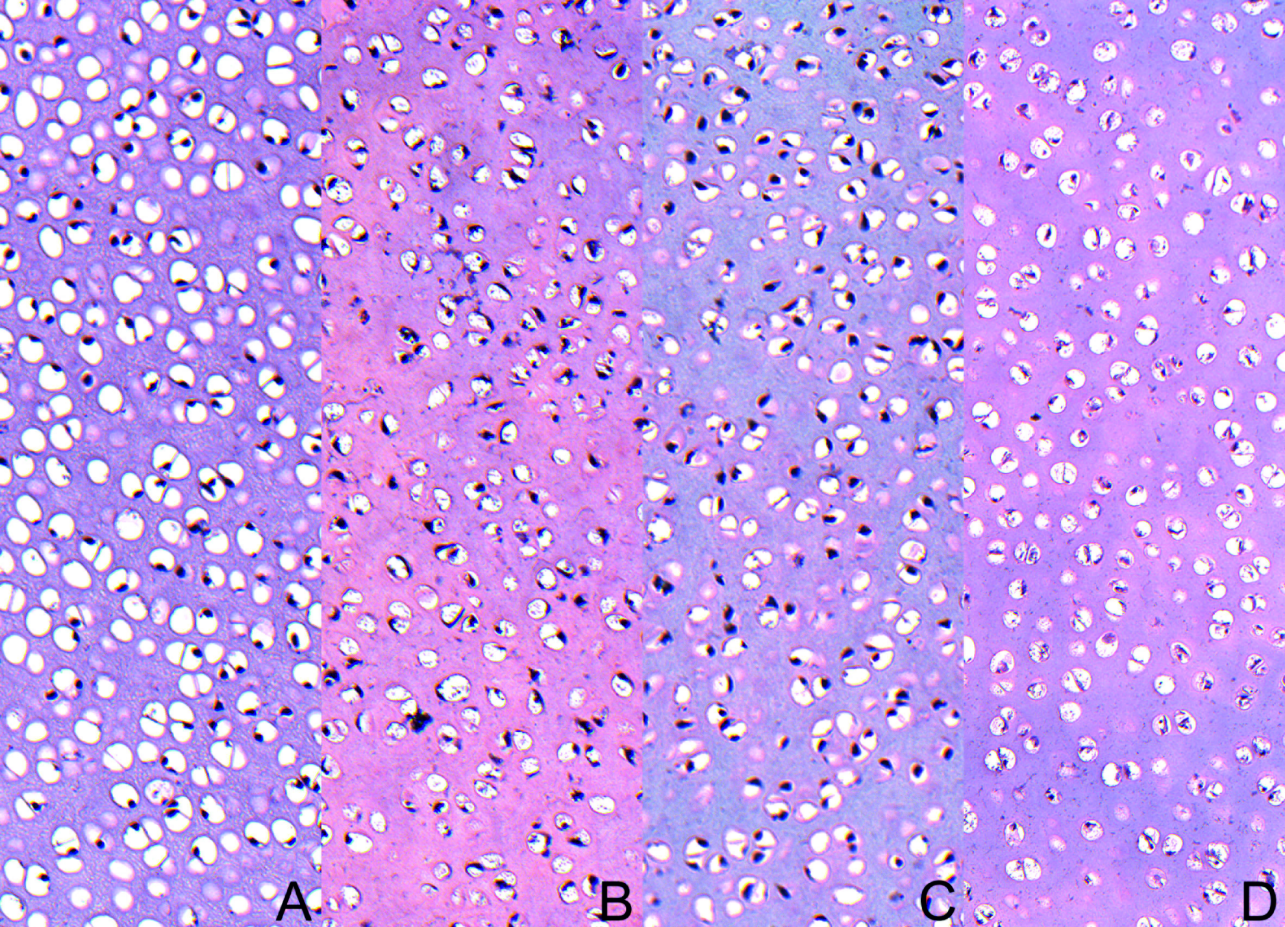
Representative images of the femoral articular cartilage stained with safranin-O carried out on formaldehyde-fixed sections. Vertical section of total femoral articular cartilage from the control male group (A), the HMB-treated male group (B), the control female group (C), and the HMB-treated female group (D). Magnification x100.

Safranine O staining showed a difference in the content of proteoglycans (colour intensity) in articular cartilage in piglets supplemented and non-supplemented with HMB through their mothers (Fig 6). Lower proteoglycan content (displaying very weak pink staining) in the cartilage from the control group of both sexes was seen, but it displayed local reaction, creating a patchwork in the cartilage matrix in the female newborns (Fig 6). In turn, the groups of both sexes after the maternal HMB treatment demonstrated moderate red-pink staining linked with higher content of proteoglycans, especially in the male newborns (Fig 7).

Even though the number of chondrocytes per square millimetre of articular cartilage was not calculated, high cellular articular cartilage was seen in the control groups of both sexes, especially in the males (Fig 7). On the other hand, a decrease in the number of chondrocytes with a high amount of articular matrix was observed in the prenatally HMB-treated animals (mainly in the female newborns).

### Differential scanning calorimetry

**Fig 8:**
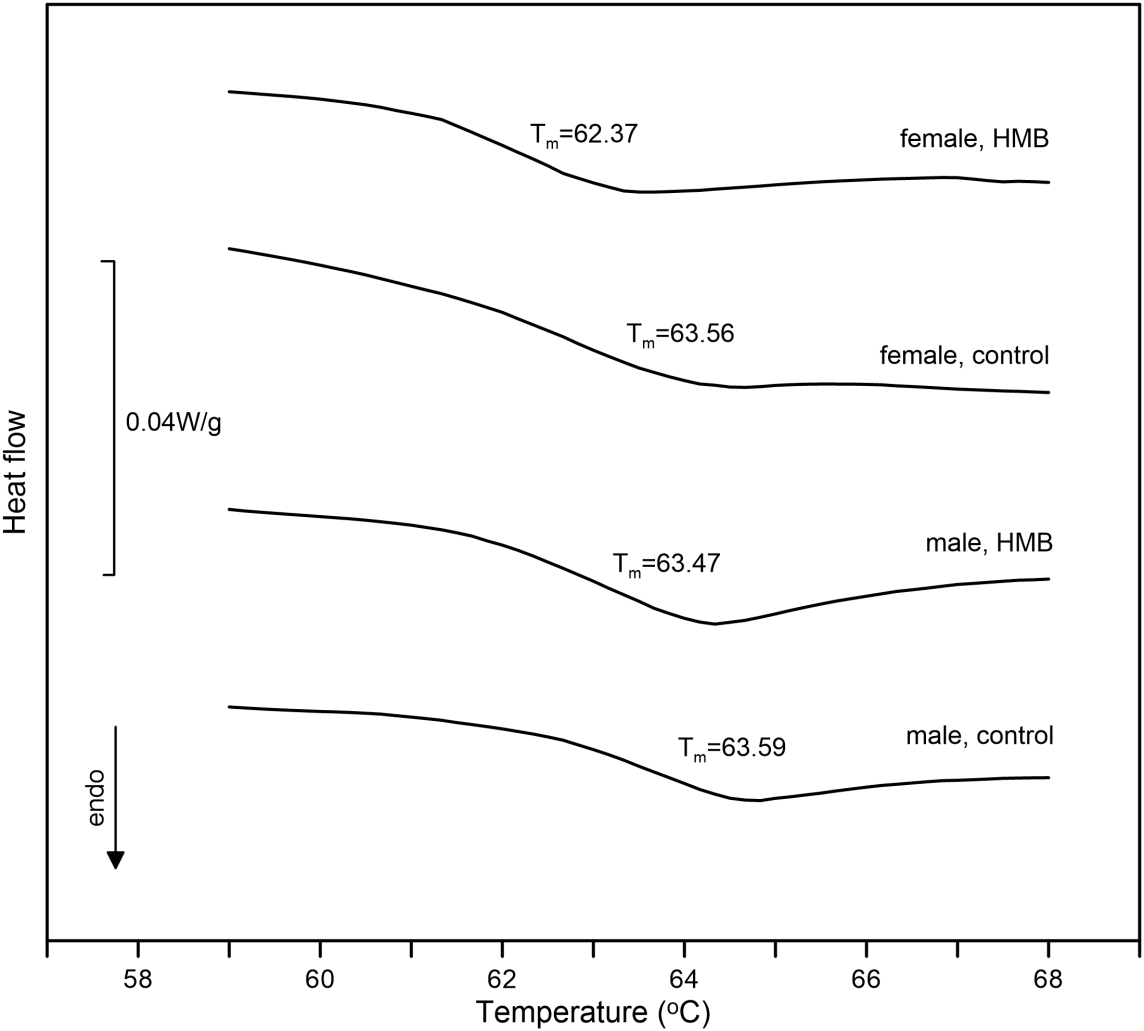
DSC thermograms of the cartilage of newborn piglets. The presented values are the mean values of calculated peak transition temperatures in each experimental group. The downward deflection of DSC scans denotes an endotherm process, indicating the denaturation of tissue collagen.

Fig 8 shows sample thermograms of cartilage tissues, and the results of thermal analysis are presented in Table 6. All the dissected samples showed a typical endothermic peak associated with irreversible denaturalization of cartilage collagen protein. The results showed that crosslinked collagen was thermally very stable in all the examined cartilage tissues, as no changes in onset temperatures were observed. The effect of HMB supplementation is manifested only in lowering the peak transition temperature in the female HMB group (T_max_ = 63.67°C).

**Table 6:** Quantitative analysis of thermal data.

Item	Female		Male		SEM	Influence of		
	control	HMB	control	HMB		treatment	sex	Interaction of treatment*sex
**Onset temperature, °C**	61.92	61.34	61.97	62.06	0.14	0.205	0.081	0.114
**Peak temperature, °C**	64.67^b^	63.67^a^	64.55^b^	64.47^b^	0.19	0.008	0.017	0.022

^a, b,^—mean values in rows with different letters differ significantly at P<0.05; SEM—standard error of the means.

## Discussion

Osteoporosis, characterized by low bone mineral density, is a common condition in elderly subjects, especially women. Because its consequences include disability and death caused by an increased risk of fractures of the hip and other bones, it is a major public health [42]. Peak bone mass, which is defined as the amount of bone tissue present at the end of the skeletal maturation, is an important determinant of the osteoporotic fracture risk. Bone strength depends on bone mass, architecture, and geometry. Other factors can also influence peak bone mass achievement, which is more or less independent. Sex, endocrine status, genetic predisposition, dietary components during prenatal time, childhood, and adolescence are determinants of bone mass and the risk of osteoporotic fractures. Our earlier studies indicated that environmental factors such as nutrition or hormonal treatment of pregnant dams program skeletal development in their offspring [7, 11, 24, 25]. Many studies indicate a significant effect of HMB on the body weight and composition caused by the anti-catabolic effect of HMB, resulting from the stimulation of its synthesis and inhibition of the degradation of muscle protein [5, 43–45].

In our study, the maternal HMB supplementation in mid-gestation through 20 days enhanced body weight of newborns only in the males (by 81%). Moreover, the weight of basal vital organs of the newborn offspring was proportional to body size (there was no difference in relative weight, data not shown). In an earlier study, HMB was given to pregnant sows in the amount of 0.05 g/kg of body weight per day, and newborns were heavier only by 23%, compared to the control group. However, the time and duration of administration (HMB was administered two weeks before delivery) differs from the scheme used in the present study. This may have been the main cause of these differences besides the dosage used. A similar effect was observed by Wan *et al*. [5], who used a diet supplemented with 4 g/day HMB-Ca from day 35 of gestation to parturition and observed a higher litter birth weight [5]. On the other hand, our other study shows a decrease in the term body weight of newborn mice depending on the time of HMB supplementation and an increase in the weight of organs that was not proportional to the body size. The supplementation with HMB significantly increases the weight of livers and kidneys. However, the livers and kidneys weight increases in newborns delivered by dams supplemented with HMB during the middle period of gestation [43]. Moreover, HMB given at the daily dosage of 0.2 g/kg of body weight in mice from day 26 of gestation until parturition caused more significant changes in the enamel surface texture and resulted in greater reduction of roughness parameters, compared to the administration of 0.02 g/kg of body weight during the same period [8]. Given this result, the dose of HMB used in the current study in sows was similar to the dose applied to the mice. The obtained results are new and the effects of HMB at the daily dosage of 0.2 g/kg of body weight on bone tissue development have not been investigated so far in any other animals and never in humans. Importantly, a common metabolic pathway of the production of endogenous HMB operates in both humans and pigs [46–49].

Although there was a big difference in the body weight after the HMB-treatment, the increase in the bone weight was the same (53% in females, 57% in males) and proportional to the body, as indicated by relative bone mass. However, the beneficial effect of the HMB-treatment was observed in males as a greater increase in bone length (by 25.6%) and bone diameter. Only 17.2% alteration of the length was noted in the supplemented females, compared to the female control group. Subsequent beneficial effects of the treatment, i.e. improved geometric and mechanical properties, were observed in the male newborns. Improved bone geometrical properties have also contributed to increased bone morphology in the males from the HMB group. The cross-sectional area, mean relative wall thickness, cortical index, and midshaft volume were by 59.8%, 101%, 38% and 100.8% higher after the HMB-treatment, while no such improvement was observed in the females. In spite of the lack of geometry improvement in the female newborns, an increase in the ultimate strength was observed (by 33.7%). However, the HMB impact on bone endurance was more marked in the male newborns. The value of ultimate strength, maximum elastic strength, and bending moment were increased by 70%, 77.6% and 122%, respectively. All these results were in contrast to those noted in the female offspring delivered by the HMB-treated sows. However, there was a similar effect of HMB on bone mineral content in both sexes. BMC increased by 97% and 83% in the male and female newborns, respectively. Probably, enhanced BMC contributed to the improved bone mechanical endurance in female newborns. The analyses of the femur showed that both the increased bone mineral content and enhanced geometrical parameters were responsible for enhanced values of mechanical parameters. Furthermore, the results obtained suggest differences in bone metabolic response to maternal HMB-treatment between the males and females. The treatment with HMB induced greater changes in compact bone in the males.

This is in contrast with another study, where HMB-treatment enhanced bone mineral density more substantially in females than in males, while the geometrical and mechanical parameters were considerably more elevated in males treated with HMB during the prenatal time. These positive effects of prenatal HMB-treatment are not linked with the difference in pigs’ body weight. The weight of the 6-month-old pigs examined in this study was similar in both sexes [7]. However, the HMB-treatment of pregnant sows for the last 14 days of the pregnancy significantly enhanced serum concentration of IGF-1 in the offspring at birth compared to the control piglets [7].

The results obtained in the current study also indicated that the positive effects of the prenatal treatment with HMB on foetal growth and development were mediated by altered function of somatotropic and pituitary-gonadal axes. This hypothesis was strongly supported by the significantly increased serum concentrations of insulin-like growth factor-1 and leptin in HMB-treated newborn piglets. The differences in the IGF-1 and leptin concentration between the investigated groups reached 166% and 33% in the females and 296% and 39% in the males, respectively. The activation of the second axis was also upregulated by the HMB-treatment, as it was supported by the concentration of FSH (a two and three fold increase in the females and males, respectively), LH, estradiol (by 122% and 310% in the females and males, respectively), and testosterone (by 33% and 42% in the females and males, respectively). These prenatally induced changes in the function of the somatotropic and pituitary-gonadal axes after the HMB-treatment in general positively influenced prenatal development of piglets, including bone metabolism. This was supported by the densitometric analysis, which showed a significant increase in BMC linked with a decreased concentration of calcium and phosphorus in blood serum. Moreover, bone metabolism was assessed by measuring serum total alkaline phosphatase, whose concentration was enhanced by the HMB-treatment by 34% in the females and 138% in the males.

This is with agreement with another study, where positive correlations between birth weight and cord blood levels of IGF-1 and leptin were found [50]. IGF-1 is a marker of secreted growth hormone and stimulates the proliferation of chondrocytes. It is also provokes osteoblasts to secret collagen and other non-collagenous proteins [50]. The main established endocrine regulators of foetal growth include leptin, besides IGF-1, a hormone secreted by adipocytes and the placenta, which is positively associated with intrauterine growth [50]. The regulation of foetal growth is complex and multifactorial. Diverse factors, including maternal and environmental factors, lead to altered intrauterine growth [51]. The mechanisms controlling intrauterine growth are incompletely understood, but an interaction of maternal, placental, and foetal endocrine factors is likely to govern partitioning of nutrients and the rate of foetal cell proliferation and maturation [50].

It is thus reasonable to speculate that the association of leptin with foetal growth mediated by IGF-I and the pituitary-gonadal axis could be the main cause of improved cell proliferation and maturation in the growth plate of our prenatally HMB-treated offspring. This was confirmed by the improved expression of the non-collagenous protein OPG (Figs 2, 3 and 4). This may indicate that the bone formation process was enhanced. Additionally, the histomorphometrical analysis showed beneficial effects of the HMB-treatment on trabecular bone. Moreover, IGF-1 was elevated in our HMB-treated piglets and it may have positively influenced the expression of the selected non-collagenous proteins.

Bone and cartilage homeostasis is regulated by GH acting via IGF-1 and the RANK/RANKL/OPG system. However, little is known how food containing a metabolite of leucine influences the expression of non-collagenous proteins. Approximately 10% of different non-collagenous proteins, the expression of which is considered to be a specific marker, are expressed by osteoblasts and chondrocytes [26, 41]. Neither the expression of the non-collagenous protein in the growth plate or trabeculae nor the alteration in the expression of OPG has been reported as well as proteoglycan content and thermal analysis of articular cartilage after nutritional modification in newborn pigs. Based on the results obtained, it can be concluded that the prenatal administration of HMB enhanced the content of proteoglycans in articular cartilage (Fig 7). The HMB-treatment exerted a protective effect on the piglets of both sexes, in which degenerative changes occurred much later, as reflected in the morphometry and the distribution of proteoglycans in the control animals (Fig 7). Improved morphology was observed in the articular cartilage in the newborns from sows supplemented with HMB during pregnancy. The increase in the thickness of the transitional zone can be beneficial, as it can influence the transfer and distribution of the load through the joint with functional consequences. The physical traits of articular cartilage are dependent on the diversity in matrix components and the difference in the quantitative ratio of collagen fibres. The physical properties of articular cartilage are determined by the diversity in the components of the matrix and the difference in the quantitative mutual relations of collagen fibres. The degradation of proteoglycans can play a pivotal role in destabilization of the collagen network. Proteoglycans and collagen fibres ensure stability and a low coefficient of friction [26]. Furthermore, the proportion of thin and thick fibres should give additional structural information about the influence of prenatal HMB supplementation on collagen synthesis in articular cartilage (Fig 5). The fine collagen fibres (green colour) restricted to the layer of calcified cartilage that interfaced articular cartilage with bone might indicate an intensive process of collagen synthesis occurring in our HMB-treated male newborns (Fig 5). Collagen is a major component of the extra-cellular matrix in many tissues, and its metabolism is directly associated with many physiological process of biological adaptations. The thermal process analysed in this study was attributed to collagen denaturation. The peak temperature of denaturation of cartilage collagen fibres was low (64°C) compared to that (73°C for human femoral condyle) reported in literature [22]. However, another study has shown that the peak temperature of denaturation of rabbit femoral trochlear is about 66°C [23]. The observed differences may have been caused by various factors. First, the collagen denaturation temperature increases with age as a result of a higher degree of collagen cross-linking, while newborn piglets were examined in our study. Secondly, the differences in collagen denaturation temperatures might result from the different conditions of analyses, i.e. different heating rates and different methods of sample preparation, compared to those provided by literature. For example, Kijowski [52] observed that soaking in a NaCl solution reduced the collagen denaturation temperature. Although our all samples were kept in physiological saline, the DSC results clearly proved that the HMB-treatment significantly changed the collagen structure in cartilages, especially in females, which was also demonstrated by the PSR analysis. The differences in the calorimetric peak temperatures between the HMB-supplemented and control females were presumably due to the different collagen fibril density in their structure (Fig 6). The cartilages from the female HMB group had morphologically less compact collagen bundles than in the control, and significantly less energy is needed to disintegrate this more loose structure (Fig 6); thus, the peak of the structural phase transformation took place at the lower temperature [53]. To the best of our knowledge, there is no published study about the influence of the prenatal nutritional programming on thermal characteristic of collagen tissues. The main advantage of this study was that the interpretation of thermal changes was confirmed by the histomorphological analysis of the cartilage structure. However, the importance of the decrease in the peak temperature noted in thermal stability analysis in this study remains to be elucidated.

In summary, this is the first report combining the histomorphometry and immunohistochemistry with DSC thermal analysis of the influence of maternal HMB supplementation on the structure and properties of cartilage tissue. The DSC analysis showed significant changes in in peak temperature in the HMB-supplemented female group. This parameter indicates thermodynamic consequences of structural rearrangement of cartilage tissue, which provides basis for further investigation of the influence of the prenatal nutritional programming on the functional performance and structure of collagen-based tissues. On the other hand, this study showed that maternal HMB supplementation in mid-gestation significantly improved the general growth and mechanical endurance of long bones by the influence on the somatotropic and pituitary-gonadal axes in the offspring.
